# The association between depressive symptoms and ischemic heart disease in postmenopausal women: a cross-sectional study

**DOI:** 10.3389/fpsyg.2025.1485291

**Published:** 2025-03-05

**Authors:** Yanyan Yuan, Gang Chen

**Affiliations:** Department of Anesthesiology, Sir Run Run Shaw Hospital, School of Medicine, Zhejiang University, Hangzhou, China

**Keywords:** National Health and Nutrition Examination Survey, depressive symptoms, ischemic heart disease, hypertension, postmenopausal women

## Abstract

**Background:**

The negative impact of depression on cardiovascular health has drawn much attention. However, the relationship between depressive symptoms and ischemic heart disease (IHD) in postmenopausal women has not been previously reported.

**Methods:**

This cross-sectional study analyzed data from the National Health and Nutrition Examination Survey (NHANES) spanning 2005 to 2018, including 6,538 postmenopausal women. Weighted multivariable logistic regression analyses were conducted to examine the independent association between depressive symptoms and IHD.

**Results:**

The fully adjusted model revealed a significant association between depressive symptoms and IHD (OR 1.97, 95% CI [1.24, 3.13]). Subgroup and interaction analyses revealed that depressive symptoms were more strongly linked to IHD risk among younger women, those with lower annual household incomes, non-Hispanic Black women, and individuals with comorbidities such as diabetes and hypercholesterolemia. Moreover, the presence of hypertension moderated the relationship between depressive symptoms and IHD.

**Conclusion:**

Our findings indicate a significant association between depressive symptoms and increased IHD prevalence among postmenopausal women in the United States, with hypertension acting as a moderating factor. These results offer new insights and potential targets for improving cardiovascular health in this population.

## Introduction

Ischemic heart disease (IHD) is defined by myocardial ischemia, hypoxia, or necrosis resulting from the narrowing or blockage of the coronary arteries. As global life expectancy rises and population age increase, IHD has become a leading cause of death and a significant public health and economic burden, ranking as the most common cardiovascular disease (CVD) (Nowbar et al., 2019) and the primary cause of mortality worldwide (World Health Organization, 2020). IHD presents differently in men and women, with notable variations in risk factors, clinical features, complications, and prognosis. Female-specific risk factors include age at menarche, polycystic ovary syndrome, infertility, early menopause, a history of preeclampsia, and use of assisted reproductive technologies (Pathak et al., 2017; Mehta et al., 2015; Zhu et al., 2019). Additionally, conditions more prevalent in women, such as autoimmune diseases, fibromyalgia, migraines, and depression, further contribute to IHD risk (Shanmugasegaram et al., 2012; Patel et al., 2020). IHD is often misdiagnosed as menopausal neurosis, delaying medical intervention. Women typically present with IHD at an older age, experience more frequent myocardial infarctions, and face a higher risk of premature death compared to men (Pathak et al., 2017; Virani et al., 2021; Wei et al., 2014). Extensive researches have shown that endogenous estrogen has a protective effect on the cardiovascular system (Mazzuca et al., 2015; Alencar et al., 2018), which helps explain the increased cardiovascular risk in postmenopausal women due to declining ovarian function and reduced estrogen levels (Sutton-Tyrrell et al., 2005).

Depressive disorder (depression) is a prevalent mental health condition marked by prolonged low mood and loss of interest or pleasure in activities. Women are more likely to experience depression than men (World Health Organization, 2023), and depressive symptoms are more frequent and severe during the menopausal transition compared to premenopausal women. Major risk factors for menopausal depression include vasomotor symptoms such as insomnia and hot flashes, a history of severe depression, and inherent susceptibility (neuroticism) (Alblooshi et al., 2023). Unlike some other conditions, menopausal depression does not necessarily resolve after menopause and may worsen over time, potentially leading to chronic conditions (Amore et al., 2007). Growing evidence links depression with an increased risk of IHD. Potential mechanisms include chronic subacute inflammation-induced arterial wall damage, lymphocyte redistribution, heightened platelet activity, endothelial dysfunction, disturbances in cardiac autonomic regulation, and hypothalamic–pituitary–adrenal axis abnormalities (Dutta et al., 2012; Morel-Kopp et al., 2009; Licht et al., 2008; Jokinen and Nordstrom, 2009). However, research on the association between depression and cardiovascular events in postmenopausal women remains limited. It is therefore crucial to investigate whether depressive symptoms independently increase the risk of IHD in postmenopausal women and whether they could be considered as a preventable prognostic factor. This study uses data from the National Health and Nutrition Examination Survey (NHANES) from 2005 to 2018 to explore the relationship between depressive symptoms and the prevalence of IHD, including coronary heart disease (CHD), angina, and heart attack in postmenopausal women.

## Materials and methods

### Study population

Data for this cross-sectional study were extracted from the NHANES (RRID: SCR_013201) database, which spans the years 2005 to 2018. Managed by the Centers for Disease Control and Prevention (CDC), the NHANES program is an ongoing initiative that assesses the health and nutritional status of both adults and children in the United States (US). Since 1999, the survey has examined a nationally representative sample of approximately 5,000 individuals annually, using a complex, multistage probability sampling design. To ensure reliable statistics, NHANES over-samples individuals aged 60 and older, as well as African Americans and Hispanics. All NHANES study protocols were reviewed and approved by the Institutional Review Board of the National Center for Health Statistics. Written informed consent was obtained from all participants, or from parents or legal guardians for those under 16. Detailed information on the NHANES study design and data is available at www.cdc.gov/nchs/nhanes/. Trained staff from the National Center for Health Statistics conducted interviews at participants’ homes and performed physical examinations at mobile centers.

**Figure 1:**
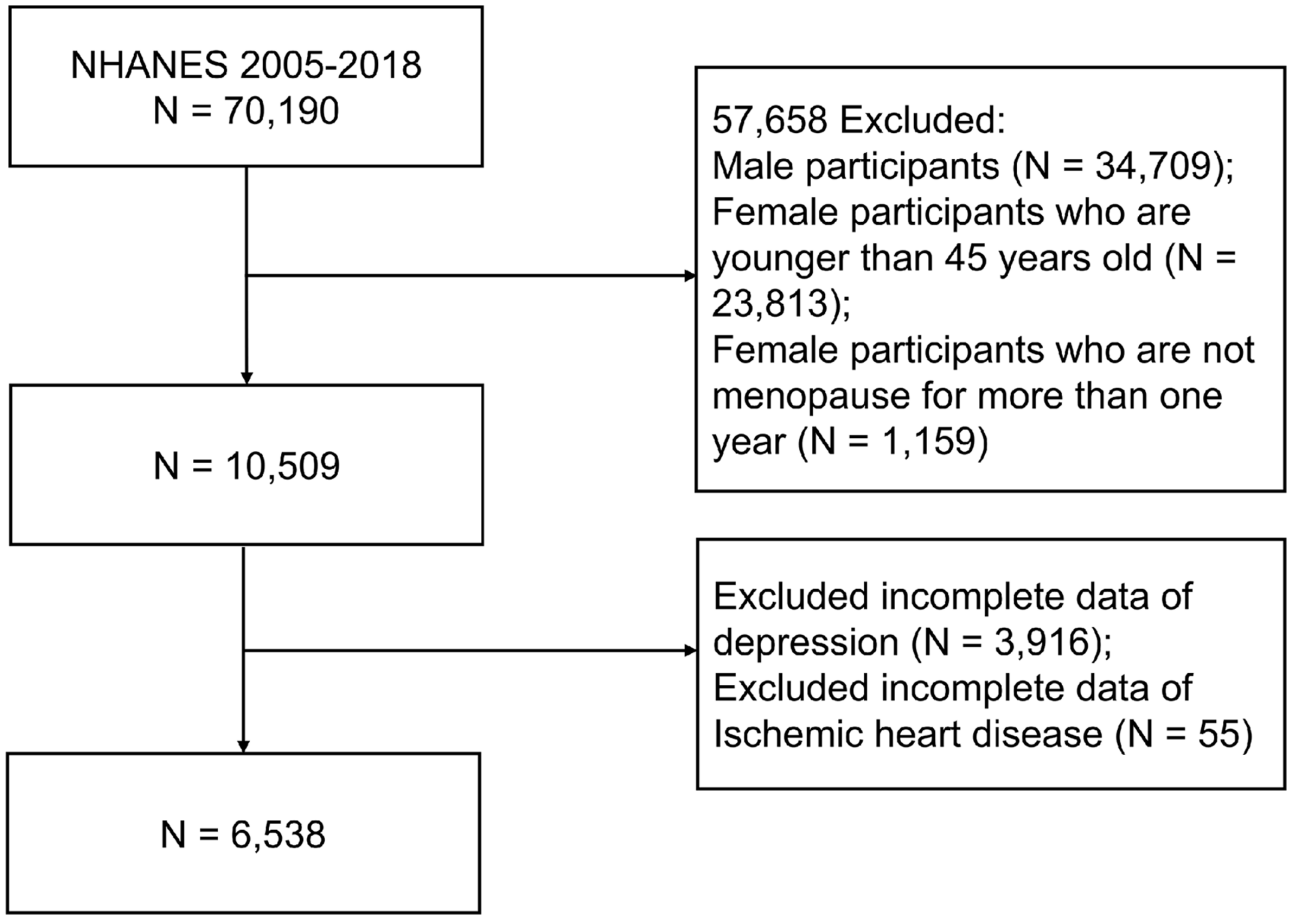
Flow chart of eligible participant selection, NHANES 2005–2018.

A total of 70,190 participants were included in the study, covering the period from 2005 to 2018. This study focused on postmenopausal women, where menopause was defined as the absence of menstrual periods for 12 consecutive months (El Khoudary et al., 2019). In the US, the median age of menopause is 52 years, with a range from 45 to 55 years (World Health Organization, 2022). Initially, we included women aged 45 and older and confirmed their menopausal status based on responses to a reproductive health questionnaire. Participants were asked, “Have you had at least one menstrual period in the last 12 months?” Participants who answered “No” were included in the study. Male participants (*N* = 34,709), female participants who are younger than 45 years (*N* = 23,813), and female participants who had not been postmenopausal for at least 1 year (*N* = 1,159) were excluded, leaving 10,509 participants. We further excluded individuals with incomplete data on depressive symptoms (*n* = 3,916) or IHD (*n* = 55). The final analysis included 6,538 participants (Figure 1). Since the data is publicly available, the Institutional Review Board at Sir Run Run Shaw Hospital, Zhejiang University School of Medicine, waived the need for additional ethical approval for this research.

### Depressive symptoms assessment

The Patient Health Questionnaire-9 (PHQ-9) is a standardized tool used to assess depression, capable of evaluating both the severity of depressive symptoms and their potential diagnostic utility. The questionnaire assesses the frequency of nine depressive symptoms experienced over the past 2 weeks. PHQ-9 scores range from 0 to 27, with each item rated on a four-point scale: 0 = “Not at all, “1 = “Several days,” 2 = “More than half the days,” and 3 = “Nearly every day.” PHQ-9 scores are categorized as follows: 0–4 (none or minimal depression), 5–9 (mild depression), 10–14 (moderate depression), 15–19 (moderately severe depression), and 20–27 (severe depression). Given the sample size of each group, moderate, moderately severe, and severe depressive symptoms were identified as the presence of depressive symptoms (Rula, n.d.). A PHQ-9 score of ≥10 demonstrated a sensitivity of 88% and a specificity of 88% for detecting major depressive disorder (Kroenke et al., 2001).

### Primary outcome and secondary outcomes

IHD was designated as the primary outcome. IHD was defined as the occurrence of any predefined secondary outcomes: CHD, angina, or heart attack (ICD LIST, 2024). Secondary outcomes were assessed during interviews using a standardized health questionnaire that asked, “Has a doctor or other health professional ever told you that you had CHD, angina, or a heart attack?” The average interval between interviews and Mobile Testing Center assessments was 2 weeks.

### Selection of covariates

Covariates in this study included age (year), race (Mexican American/Other Hispanic/Non-Hispanic White/Non-Hispanic Black/Other races), education level (less than high school/high school or equivalent/college or higher), marital status (single/married), annual household income (< $2000/ ≥$2000), body mass index (BMI, kg/m^2^), diabetes (yes/no), hypertension (yes/no), and hypercholesterolemia (yes/no). Smoking status was determined through household interviews and categorized into those who had smoked fewer than 100 cigarettes in life versus those who had smoked 100 or more in life. Alcohol consumption was categorized as ≤1 drink per day or > 1 drink per day, with one drink defined as a 12 oz. beer, a 5 oz. glass of wine, or 1.5 oz. of liquor. Marital status categories included “single” (widowed, divorced, separated, never married, or cohabitating with a partner). Detailed procedures for these measurements are available at www.cdc.gov/nchs/nhanes/.

### Statistical analyses

Statistical analyses followed Centers for Disease Control and Prevention guidelines, incorporating NHANES sampling weights to account for the complex multi-stage cluster sampling design. Continuous variables were summarized as means with standard deviations (SD), and categorical variables were presented as proportions. Univariate and multivariate logistic regression analyses were used to examine the association between depressive symptoms and IHD across three models. PHQ-9 scores were analyzed as both continuous and binary variables, with scores ≥10 indicating depressive symptoms. Model 1 was unadjusted, serving as the crude model. Model 2 was adjusted for age and race. Model 3 was further adjusted for age, race, education level, marital status, annual household income, BMI, diabetes, hypertension, hypercholesterolemia, smoking status, and alcohol consumption. Subgroup analyses explored the relationship of depressive symptoms and IHD across categories such as age, race, annual household income, diabetes, hypertension, and hypercholesterolemia. These stratification factors were evaluated as potential effect modifiers. Interaction terms were applied to assess heterogeneity in associations among subgroups. All analyses were conducted using Empower software (www.empowerstats.com; X&Y Solutions, Inc., Boston, MA), with a significance threshold of *p* < 0.05.

## Results

### Baseline characteristics of participants

Table 1 summarizes the characteristics of 6,538 participants, stratified by IHD presence. The prevalence of IHD among postmenopausal women was 9.42%. Higher IHD risk was linked to older age, less education, lower household income, being unmarried, greater smoking and alcohol consumption, and higher obesity levels. Participants with IHD were also more likely to have comorbid conditions, including diabetes, hypertension, and hypercholesterolemia. Depressive symptoms were present in 19.71% of individuals with IHD, more than double the rate in those without IHD.

**Table 1:** Weighted baseline characteristics of the study population by ischemic heart disease, NHANES 2005–2018 (*N* = 6,538).

	Non-ischemic heart disease^a^	Ischemic heart disease^a^	*p*-value
Number of participants (%)	5,922 (90.58)	616 (9.42)	
Age (%)			<0.0001^b^
<=55	1,311 (22.13)	45 (7.27)	
>55, <=65	2,164 (36.54)	166 (26.88)	
>65	2,448 (41.34)	406 (65.85)	
Race (%)			0.8482^b^
Mexican American	297 (5.01)	25 (3.98)	
Other Hispanic	247 (4.17)	26 (4.27)	
Non-Hispanic White	4,433 (74.85)	461 (74.84)	
Non-Hispanic Black	614 (10.37)	67 (10.84)	
Other races	332 (5.61)	37 (6.07)	
Education level (%)			<0.0001^b^
<high school	862 (14.56)	138 (22.39)	
High school	1,525 (25.75)	195 (31.63)	
>high school	3,535 (59.69)	283 (45.98)	
Marital status			<0.0001^b^
Single	2,581 (43.58)	370 (60.04)	
Married	3,341 (56.42)	246 (39.96)	
Annual household income (%)			<0.0001^b^
<$20,000	1,428 (24.12)	241 (39.14)	
> = $20,000	4,494 (75.88)	375 (60.86)	
Alcohol consumption (%)			0.0437^b^
<=1	3,458 (58.39)	398 (64.64)	
>1	2,464 (41.61)	218 (35.36)	
Smoking status (%)	2,398 (40.50)	340 (55.12)	<0.0001^b^
Diabetes (%)	3,778 (63.80)	525 (85.22)	<0.0001^b^
Hypertension (%)	4,982 (84.13)	560 (90.86)	0.0003^b^
Hypercholesterolemia (%)	3,010 (50.82)	435 (70.62)	<0.0001^b^
Depression symptoms	528 (8.92)	121 (19.71)	<0.0001^b^
Body mass index (kg/m^2^)	29.67 ± 7.28	30.34 ± 7.34	0.0411^c^

^aValues are mean ± standard deviation for continuous variables and percentages for categorical variables.^

^bp value was calculated by weighted chi-square test.^

^cp value was calculated by weighted linear regression model.^

Supplementary Table S1 details the characteristics of 6,538 participants, categorized by the presence of depressive symptoms. Among postmenopausal women, the incidence of depressive symptoms was 11.69%. Significant demographic differences were noted between participants with and without depressive symptoms. Individuals with depressive symptoms were younger, less educated, had lower household incomes, were more likely to be unmarried, and exhibited higher smoking, alcohol use, and obesity levels. They also had a higher prevalence of diabetes and hypercholesterolemia. Non-Hispanic white individuals had a lower incidence of depressive symptoms. The occurrence of depressive symptoms seemed to be unrelated to the presence of hypertension. Additionally, individuals with depressive symptoms showed a higher proportion of IHD.

### Depressive symptoms and increased prevalence of IHD

Table 2 summarizes the logistic regression analysis results examining the relationship between depressive symptoms and IHD. The fully adjusted model demonstrated a positive association between depressive symptoms and IHD. The multivariable-adjusted odds ratio (OR) with 95% confidence intervals (CI) was 1.97 (1.24, 3.13). Analysis of secondary outcomes revealed a significant association between depressive symptoms and CHD (OR 2.59, 95% CI [1.40, 4.79]) and angina (OR 1.93, 95% CI [1.03, 3.62]). However, no significant association was found between depressive symptoms and heart attacks. These results are visualized in a forest plot (Supplementary Figure S1). The association remained significant in the sensitivity analysis, where the PHQ-9 scores were analyzed as a continuous variable (Table 2). The OR (95% CI) for the PHQ-9 scores and IHD was 1.07 (1.03, 1.11), indicating that each one-point increase in PHQ-9 scores corresponded to a 7% increase in IHD prevalence. In secondary outcomes analysis, PHQ-9 scores were positively associated with CHD, angina, and heart attacks, with the strongest relationship observed for CHD.

**Table 2:** Weighted OR (95% CI) of ischemic heart disease and secondary outcomes (coronary heart disease, heart attack, and angina) across depressive symptoms among postmenopausal women in the US from 2005–2018.

	PHQ-9 scores		Depressive symptoms (PHQ > =10)	
	Multivariable^a^ logistic regression OR (95% CI)^b^	*p*-value	Multivariable^a^ logistic regression OR (95% CI)^b^	*p*-value
Ischemic heart disease	1.07 (1.03, 1.11)	0.0001	1.97 (1.24, 3.13)	0.0043
Secondary outcomes				
Coronary heart disease	1.09 (1.04, 1.14)	0.0003	2.59 (1.40, 4.79)	0.0025
Angina	1.07 (1.02, 1.12)	0.0028	1.93 (1.03, 3.62)	0.0403
Heart attack	1.05 (1.00, 1.10)	0.0585	1.73 (0.92, 3.26)	0.0917

^aAdjusted for age, race, educational level, marital status, annual household income, smoking status, alcohol consumption, diabetes, hypertension, hypercholesterolemia, and body mass index.^

^b^OR (95% CI) for primary outcome (ischemic heart disease) and secondary outcomes (coronary heart disease, angina, and heart attack) in multivariable logistic regression.

PHQ, Patient Health Questionnaire.

### Subgroup analysis

**Figure 2:**
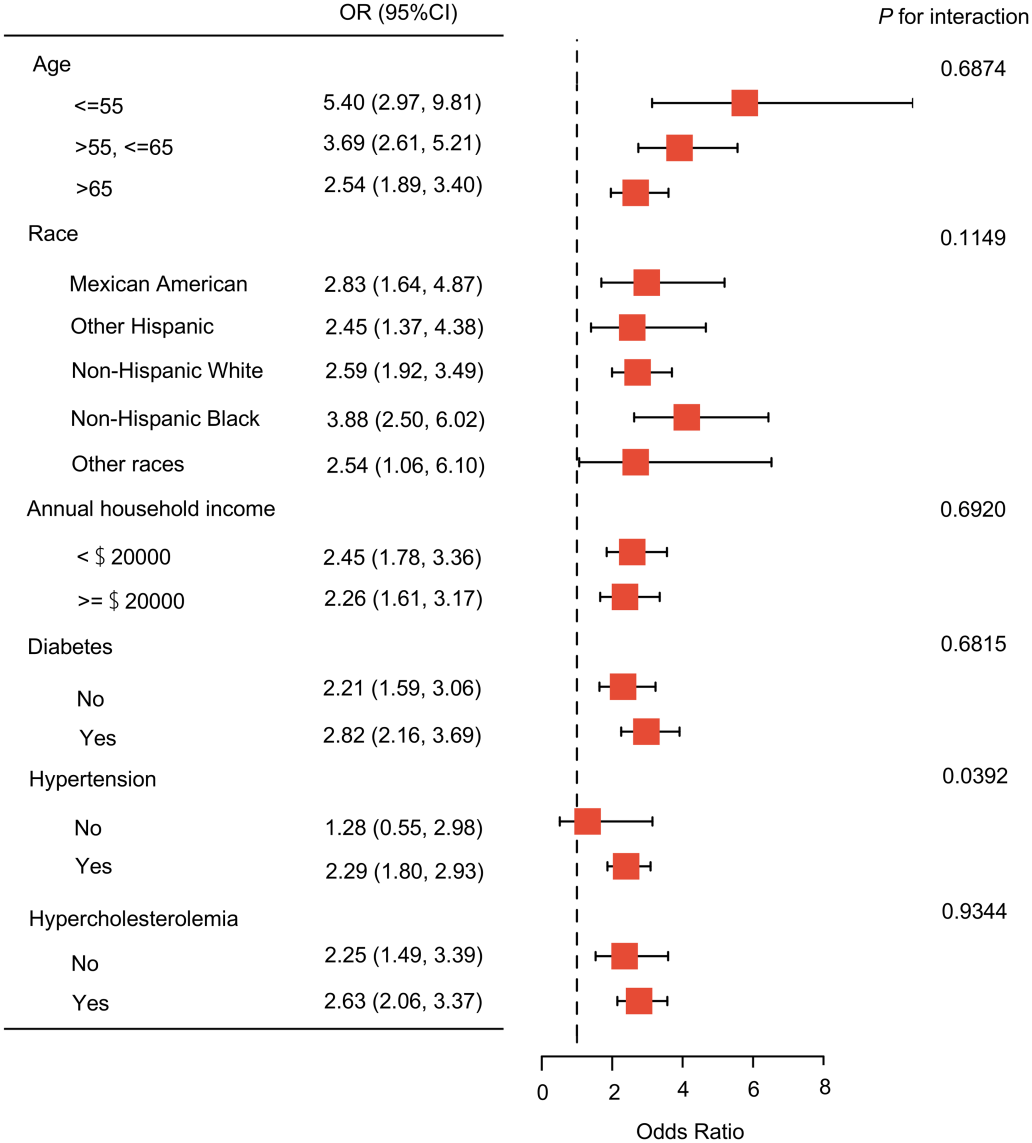
The stratified and interaction analysis of the relationship between depressive symptoms and ischemic heart disease among postmenopausal women in the US from 2005 to 2018. Adjusted for age, race, educational level, marital status, annual household income, smoking status, alcohol consumption, diabetes, hypertension, hypercholesterolemia, and body mass index, except the variable itself. *p* < 0.05 was considered statistically significant. OR (95% CI), Odds Ratio and 95% Confidence Intervals.

Figure 2 illustrates a subgroup analysis stratified by age, race, annual household income, diabetes, hypertension, and hypercholesterolemia. The stratified analysis revealed a significant association between depressive symptoms and IHD across all subgroups, with no significant interaction effects (*P* for interaction >0.05), except for hypertension. The association between depressive symptoms and IHD was stronger in younger individuals. Among racial groups, depressive symptoms were most strongly associated with IHD in non-Hispanic Black participants. No significant relationship was observed between depressive symptoms and IHD in postmenopausal women without hypertension. Conversely, in postmenopausal women with hypertension, depressive symptoms were positively correlated with IHD, as well as the second outcomes (CHD, angina, and heart attack) (Supplementary Figure S2). This association showed an interaction effect with hypertension (*P* for interaction =0.0392).

**Figure 3:**
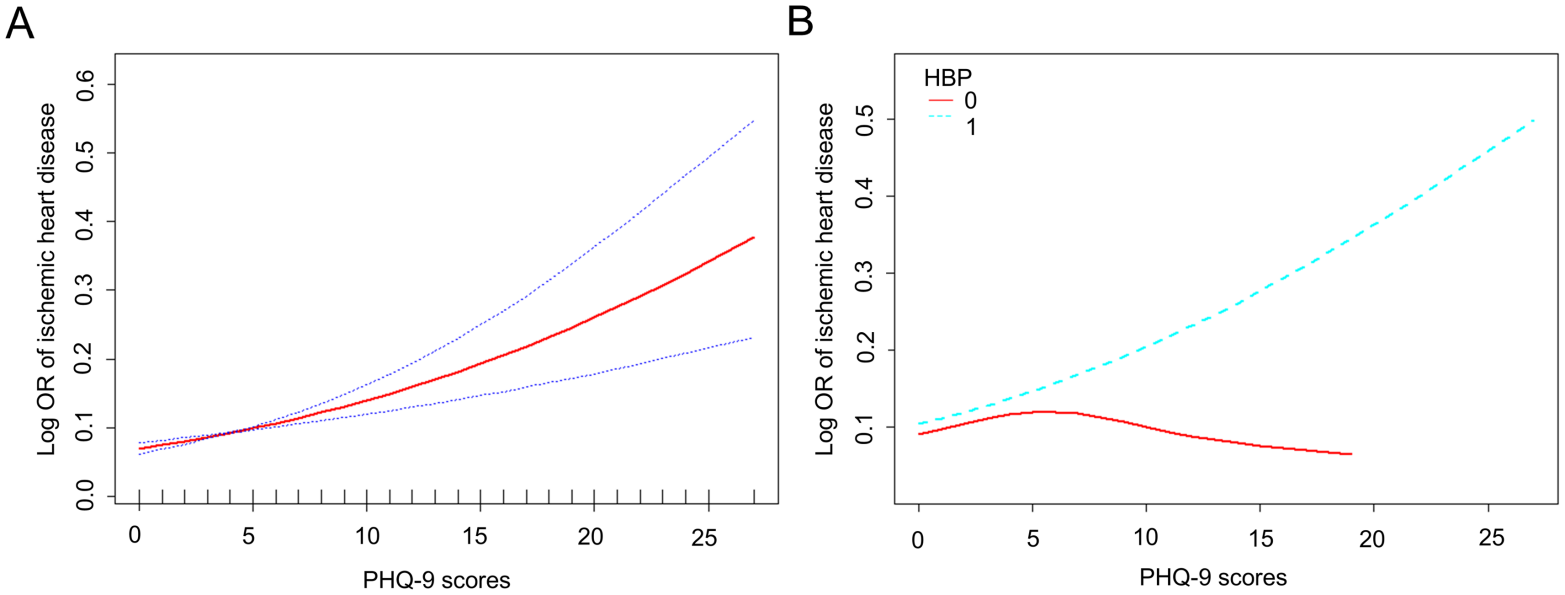
Smooth curve fitting for the relationship between PHQ-9 scores and ischemic heart disease among postmenopausal women with different hypertension status in the US from 2005–2018. **(A)** Smooth curve fitting visualizing the association between PHQ-9 scores and log-transformed odds ratio of ischemic heart disease based on the generalized additive model among postmenopausal women. The red line represents the fitted line and the blue line refers to the 95% confidence intervals. Adjusted for age, race, educational level, marital status, annual household income, smoking status, alcohol consumption, diabetes, hypertension, hypercholesterolemia, and body mass index. **(B)** Smooth curve fittings model in the subgroup analysis stratified by hypertension status: the red line denotes participants without hypertension, while the blue line denotes those with hypertension. Adjusted for age, race, educational level, marital status, annual household income, smoking status, alcohol consumption, diabetes, hypercholesterolemia, and body mass index. PHQ, Patient Health Questionnaire; HBP, High Blood Pressure, OR, Odds Ratio.

The dose–response relationship between PHQ-9 scores and IHD risk was assessed using PHQ-9 scores as a continuous variable. In the fully adjusted model, a smooth curve fit revealed an approximately linear association between PHQ-9 scores and IHD prevalence (Figure 3). However, after stratifying by hypertension status, the near-linear relationship between PHQ-9 scores and IHD was observed only in postmenopausal women with hypertension.

## Discussion

This cross-sectional study of 6,538 subjects identified a positive correlation between depressive symptoms and IHD in postmenopausal women in the US, independent of confounding factors such as age, race, income, diabetes, and hypercholesterolemia, but influenced by hypertension. Baseline data revealed a significant increase in IHD prevalence among postmenopausal women over 65 years. The excess IHD risk linked to depressive symptoms was more pronounced in younger postmenopausal women, consistent with the age distribution of depressive symptoms in this group. Although depressive symptoms are connected to various causes of mortality, few clinical trials have investigated their impact on the timing of cardiovascular events. Our results emphasize the potential need for screening depression and depressive symptoms in menopausal women, which may facilitate early identification and management of women at high risk of CVD.

Liu et al. (2022) conducted a cross-sectional study involving 29,050 participants, identifying a correlation between depressive symptoms and an increased risk of IHD in American adults. However, they reported that depressive symptoms did not significantly affect IHD in menopausal women, which is inconsistent with our findings. We reviewed the supplementary documents of that study and found that angina was associated with an increased risk of depression (OR 5.37, 95% CI [1.71, 16.88]) in menopausal women. Differences in sample size (2005–2018 vs. 2007–2018 datasets) and the inclusion of postmenopausal rather than menopausal women may explain the differing outcomes. A summary analysis of 22 prospective studies (Harshfield et al., 2020) revealed that baseline depressive symptoms are linked to the incidence of CVD, even in individuals with subclinical depressive symptoms. Additionally, the link between depressive symptoms and cardiovascular risk cannot be fully explained by known or emerging risk factors. In our study, depressive symptoms did not appear to influence hypertension development in postmenopausal women, with hypertension prevalence approximately 85% in both groups. This prevalence aligns with rates reported for older adult individuals in the NHANES database (Whelton et al., 2018). However, this rate is somewhat higher than that reported in clinical guidelines, which may be because the diagnosis of hypertension in the NHANES database was based on a single visit or a single blood pressure measurement. In this study, depressive symptoms were associated with a higher IHD prevalence only when co-occurring with hypertension. There might be a synergistic effect between hypertension and Depressive symptoms. Overlapping pathophysiological pathways of depression and hypertension may partially explain this phenomenon. The primary mechanism involves excessive sympathetic nervous system activity. Sympathetic activation is a distinct feature of primary hypertension (Goldstein and Lake, 1984), and depression is commonly associated with autonomic nervous system abnormalities, particularly heightened sympathetic activity and inadequate vagal regulation. Depressed individuals exhibit elevated norepinephrine levels in plasma, urine, and cerebrospinal fluid (de Villiers et al., 1987), accompanied by reduced heart rate variability (Carney et al., 1995). Periodic norepinephrine surges, followed by depletion, may reduce appetite, sleep, and physical activity in depressed individuals. This increased susceptibility to hypertension may exacerbate pathogenic blood pressure variability, promoting coronary artery damage, plaque formation, plaque rupture, and acute coronary events (Siever and Davis, 1985). Furthermore, depression and hypertension may share underlying genetic vulnerabilities. Reduced hypothalamic–pituitary–adrenal axis reactivity has been linked to learned helplessness (Edwards et al., 2000), which angiotensin-converting enzyme inhibitors (Martin et al., 1990) may counteract. This indicates that the renin-angiotensin system may influence both hypertension and depression, though the mechanisms remain unclear. Antidepressants may elevate blood pressure persistently or cause orthostatic hypotension (Amsterdam et al., 1999; Scalco et al., 2000), complicating blood pressure management in hypertensive patients and increasing cardiovascular risk. Stewart et al. (2023) found that a 12-month eIMPACT intervention, including internet-and telephone-based cognitive behavioral therapy, and/or collaborative care with antidepressants, was more effective for treating depression before clinical CVD onset than conventional care. However, this approach did not sufficiently reduce the heightened cardiovascular risk linked to depression. Our study suggests that combining medical and psychological interventions for depression with improved adherence to hypertension treatment, along with careful monitoring and adjustment of related medications, may more effectively achieve the desired cardiovascular benefits.

This study’s strength lies in its inclusion of a nationally representative sample of 100,000 non-hospitalized U.S. citizens across all 50 states and the District of Columbia. All analyses accounted for NHANES sampling weights and included adjustments for known IHD risk factors. The association between depression and IHD was assessed using both depressive symptoms and PHQ-9 scores, enhancing the robustness of the findings. However, several limitations should be noted. First, the cross-sectional design prevents causal inferences. Second, hypertension diagnosis relied on self-reported data, potentially introducing recall bias. Additionally, the lack of information on blood pressure variability limits the ability to further analyze whether the positive correlation between depressive symptoms and IHD in hypertensive individuals is attributable to the effects of depressive symptoms on hypertension control. Third, unmeasured or inaccurately measured confounding factors, such as baseline comorbidities, may not have been fully accounted for. Finally, the analysis included only participants with complete risk factor data, potentially reducing efficiency and introducing selection bias.

### Conclusion

Our findings reveal a significant association between depressive symptoms and increased prevalence of IHD among postmenopausal women in the US, with the strongest link observed in CHD cases. This relationship is influenced by the presence of hypertension but appears unaffected by diabetes or hypercholesterolemia. We believe that special clinical attention should be directed toward women who are particularly affected by neurovegetative symptoms during the menopausal transition, especially those with concurrent hypertension. Reducing cardiovascular events in postmenopausal women may be achievable through comprehensive assessments, targeted medical interventions, and improved self-management strategies. Future randomized controlled trials and cohort studies are essential to validate these findings and to refine prevention and treatment approaches for this vulnerable population.

## Data Availability

The original contributions presented in the study are included in the article/Supplementary material, further inquiries can be directed to the corresponding author.

## Supplementary material

SUPPLEMENTARY FIGURE S1 The forest plot depicts the OR (95% CI) of the association between depressive symptoms and ischemic heart disease among postmenopausal women in the US from 2005 – 2018. Adjusted for age, race, educational level, marital status, annual household income, smoking status, alcohol consumption, diabetes, hypertension, hypercholesterolemia, and body mass index.

SUPPLEMENTARY FIGURE S2 The forest plot depicts the relationship between depressive symptoms depressive symptoms with ischemic heart disease and secondary outcomes (coronary heart disease, heart attack, and angina) among postmenopausal women in the US, stratified by hypertension status. Adjusted for age, race, educational level, marital status, annual household income, smoking status, alcohol consumption, diabetes, hypertension, hypercholesterolemia, and body mass index.

SUPPLEMENTARY TABLE S1 Weighted baseline characteristics of the study population by depression symptoms, NHANES 2005 – 2018 (*N* = 6,538).
