# Supplementary figures and images for: The association between depressive symptoms and ischemic heart disease in postmenopausal women: a cross-sectional study
Source: Front Psychol. 2025 Mar 5;16:1485291. doi: 10.3389/fpsyg.2025.1485291 (PMC11920154; doi:10.3389/fpsyg.2025.1485291)

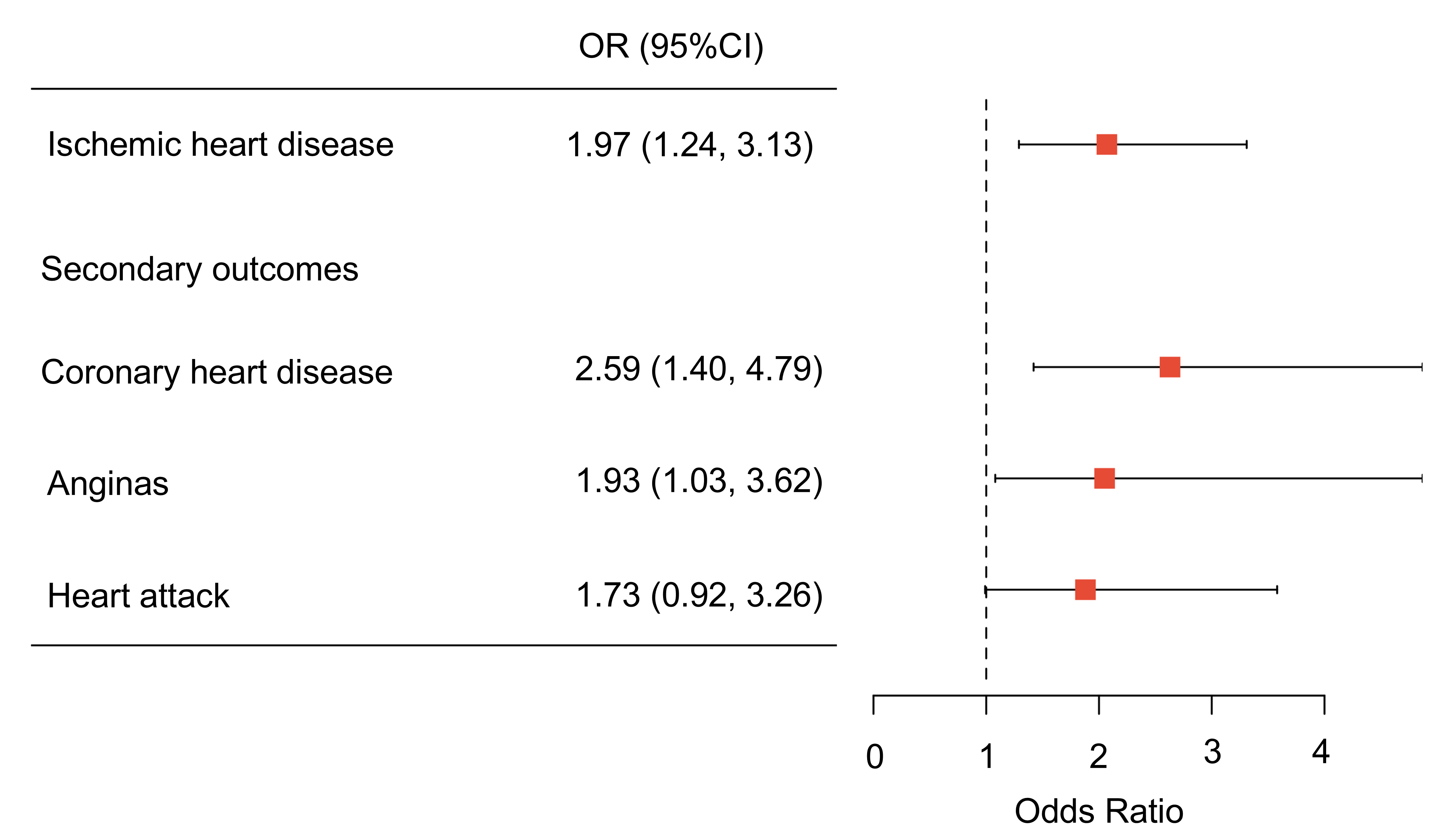

Supplement: SUPPLEMENTARY FIGURE S1 — The forest plot depicts the OR (95% CI) of the association between depressive symptoms and ischemic heart disease among postmenopausal women in the US from 2005 – 2018. Adjusted for age, race, educational level, marital status, annual household income, smoking status, alcohol consumption, diabetes, hypertension, hypercholesterolemia, and body mass index. [file Image_1.tif]

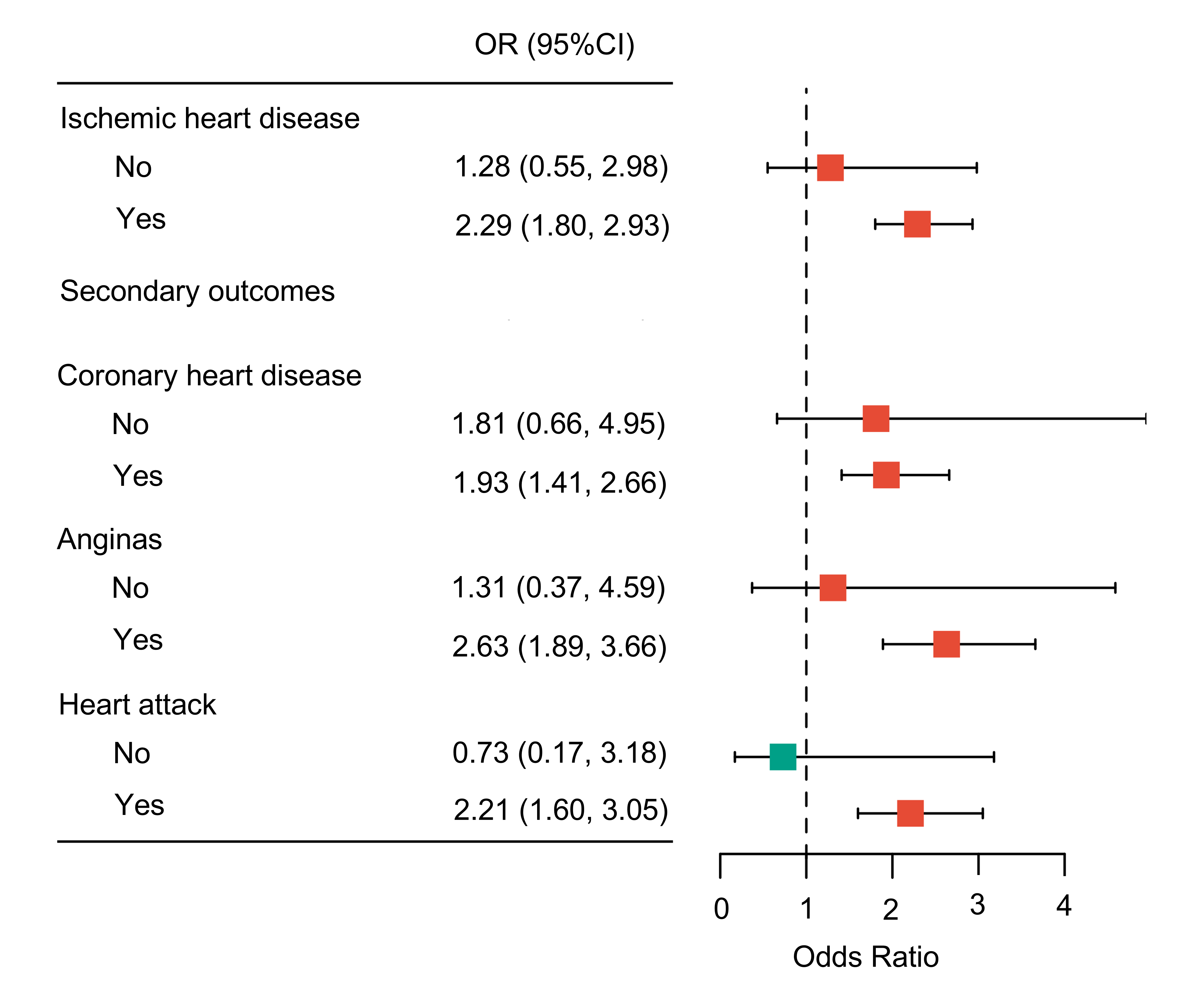

Supplement: SUPPLEMENTARY FIGURE S2 — The forest plot depicts the relationship between depressive symptoms depressive symptoms with ischemic heart disease and secondary outcomes (coronary heart disease, heart attack, and angina) among postmenopausal women in the US, stratified by hypertension status. Adjusted for age, race, educational level, marital status, annual household income, smoking status, alcohol consumption, diabetes, hypertension, hypercholesterolemia, and body mass index. [file Image_2.tif]
