# Supplementary material for: The association between depressive symptoms and ischemic heart disease in postmenopausal women: a cross-sectional study
Source: Front Psychol. 2025 Mar 5;16:1485291. doi: 10.3389/fpsyg.2025.1485291 (PMC11920154; doi:10.3389/fpsyg.2025.1485291)
Supplement: SUPPLEMENTARY TABLE S1 — Weighted baseline characteristics of the study population by depression symptoms, NHANES 2005 – 2018 (N = 6,538). [file Table_1.docx]

|  | Without- depressive symptoms（PHQ<10）^a^ | | Depressive symptoms (PHQ>=10)^a^ | *P*-value |
| --- | --- | --- | --- | --- |
| Number of participants (%) | 5774(88.31) | | 764 (11.69) |  |
| Age (%) |  | |  | <0.0001^b^ |
| <=55 | 1165 (20.18) | | 209 (27.30) |  |
| >55, <=65 | 2049 (35.48) | | 290 (37.97) |  |
| >65 | 2560 (44.34) | | 265 (34.73) |  |
| Race (%) |  | |  | <0.0001^b^ |
| Mexican American | 263 (4.56) | | 63 (8.23) |  |
| Other Hispanic | 227 (3.93) | | 49 (6.42) |  |
| Non-Hispanic White | 4374 (75.76) | | 508 (66.51) |  |
| Non-Hispanic Black | 594 (10.29) | | 88 (11.46) |  |
| Other races | 315 (5.46) | | 56 (7.39) |  |
| Education level (%) |  | |  | <0.0001^b^ |
| <high school | 805 (13.95) | | 205 (26.82) |  |
| High school | 1494 (25.87) | | 226 (29.62) |  |
| >high school | 3474 (60.17) | | 333 (43.57) |  |
| Marital status | |  |  | <0.0001^b^ |
| Single | 2508 (43.43) | | 451 (59.06) |  |
| Married | 3266 (56.57) | | 313 (40.94) |  |
| Annual household income (%) | | |  | <0.0001^a^ |
| <＄20000 | 1345 (23.29) | | 319 (41.81) |  |
| >=＄20000 | 4429 (76.71) | | 445 (58.19) |  |
| Alcohol consumption (%) |  | |  | 0.0011^b^ |
| <=1 | 3439 (59.56) | | 384 (50.24) |  |
| >1 | 2335 (40.44) | | 380 (49.76) |  |
| Smoking status (%) | 2323 (40.24) | | 423 (55.35) | <0.0001^b^ |
| Diabetes (%) | 882 (15.28) | | 219 (28.68) | <0.0001^b^ |
| Hypertension (%) | 4892 (84.73) | | 662 (86.61) | 0.3066^b^ |
| Hypercholesterolemia (%) | 2961 (51.28) | | 489 (63.99) | <0.0001^b^ |
| Ischemic heart disease (%) | 432 (7.48) | | 129 (16.84) | <0.0001^b^ |
| Coronary heart disease (%) | 240 (4.16) | | 68 (8.86) | <0.0001^b^ |
| Angina (%) | 168 (2.91) | | 55 (7.16) | <0.0001^b^ |
| Heart attack (%) | 221 (3.82) | | 62 (8.16) | <0.0001^b^ |
| Body mass index (kg/m^2^) | 29.47 ± 7.13 | | 32.04 ± 8.26 | <0.0001^c^ |

^a^ Values are mean ± standard deviation for continuous variables and percentages for categorical variables.

^b^ *P* value was calculated by weighted chi-square test.

^c^ *P* value was calculated by weighted linear regression model.
